# Radical nephrectomy with inferior vena cava tumor thrombectomy in Mayo III–IV renal cell carcinoma: a retrospective single-center study

**DOI:** 10.3389/fsurg.2025.1644948

**Published:** 2025-11-13

**Authors:** Alexey Shabunin, Dmitry Grekov, Alexey Karpov, Pavel Drozdov, Yuri Karabach, Dmitry Levikov, Igor Glushenko, Zurab Bagatelia, Evgeny Veliev, Andrey Bogdanov

**Affiliations:** 1 GBUZ Gorodskaa Kliniceskaa Bol'nica Imeni S P Botkina Departamenta Zdravoohranenia Goroda Moskvy, Moscow, Russia; 2 Rossijskaa Medicinskaa Akademia Nepreryvnogo Professional'nogo Obrazovania, Moscow, Russia; 3 Department of Health, Moscow, Russia

**Keywords:** renal cell carcinoma (RCC), inferior vena cava (IVC), tumor thrombus, radical nephrectomy (RN), cardiopulmonary bypass, Mayo classification, surgical oncology, long-term survival

## Abstract

**Background:**

Tumor thrombus extending into the inferior vena cava (IVC) in patients with renal cell carcinoma (RCC), particularly at Mayo levels III and IV, presents a major surgical challenge. Although systemic treatments are evolving, surgery remains the mainstay of management. The role of cardiopulmonary bypass (CPB) in this setting is not clearly defined.

**Methods:**

We retrospectively analyzed 20 patients with RCC and Mayo level III–IV IVC tumor thrombus who underwent radical nephrectomy with IVC thrombectomy at our center between 2017 and 2024. Preoperative workup included MRI, contrast-enhanced CT, and transthoracic/transesophageal echocardiography. CPB was used selectively in five patients with tumor extension into and adherence to the right atrium. Postoperative complications were classified using the Clavien–Dindo system. Survival was assessed with Kaplan–Meier analysis and Cox regression.

**Results:**

Median age was 61 years (IQR 51–72), and 70% were male. Level IV thrombus was present in 60% of patients, and 40% had distant metastases. Median operative time was 370 minutes and median blood loss was 2,500 mL. Postoperative complications occurred in 20% of patients, with one in-hospital death (5%). Median hospital stay was 11 days. The 1-, 3-, and 5-year overall survival rates were 66.7%, 41.6%, and 34.6%, respectively. Distant metastases were associated with lower survival (HR 2.48; *p* = 0.005), while immuno-targeted therapy improved outcomes (HR 0.69; *p* = 0.035).

**Conclusion:**

Radical nephrectomy with IVC thrombectomy in patients with advanced tumor thrombus can be performed safely with good long-term outcomes in selected cases. Careful preoperative imaging, intraoperative echocardiography, and the selective use of CPB are key to minimizing risks. These findings support a tailored surgical approach based on thrombus level and clinical condition. Further prospective studies are needed to refine surgical indications and clarify the role of systemic therapy.

## Introduction

Renal cell carcinoma (RCC) makes up 2%–3% of all adult malignancies and is the most common type of kidney cancer. In 2020, over 400,000 new RCC cases were diagnosed globally, with a reported 5-year overall survival of 77.6% (1). Established risk factors include hypertension, obesity, smoking, and chronic kidney disease (2).

A hallmark of RCC is its ability to invade the venous system, most often the renal vein and inferior vena cava (IVC). Tumor thrombus involving the IVC occurs in 4%–10% of patients, and in about 1% of cases, the thrombus extends into the right atrium. This finding is associated with more aggressive disease and reduced survival (3).

The Mayo Clinic classification is widely used to define the extent of tumor thrombus and guide surgical planning (4). This system describes five levels of thrombus extension:

• Level 0: thrombus confined to the renal vein;
• Level I: thrombus extends ≤2 cm into the IVC above the renal vein ostium;
• Level II: thrombus extends >2 cm into the IVC but remains below the hepatic veins;
• Level III: thrombus reaches or extends above the hepatic veins but remains infra-diaphragmatic;
• Level IV: thrombus extends above the diaphragm, including cases with right atrial involvement.
• Level IV thrombi present the greatest surgical challenge and are associated with increased intraoperative risk and poorer oncologic outcomes (5).

Despite advances in systemic therapies, radical nephrectomy with IVC thrombectomy remains the main treatment for patients with RCC and venous tumor thrombus, particularly at Mayo levels III and IV (6). These procedures are technically demanding and carry a substantial risk of perioperative complications. Perioperative mortality has been reported to range from 5% to 10% in large retrospective cohorts (4, 7, 8).

The decision to use cardiopulmonary bypass (CPB) during surgery remains controversial, especially in cases of level IV thrombus (9). Although clinical experience has grown substantially, no universally accepted guidelines exist for the management of RCC with extensive venous involvement, highlighting the need for further investigation (10).

This case series evaluates perioperative and long-term oncologic outcomes in patients undergoing radical nephrectomy with IVC thrombectomy for RCC with Mayo level III–IV thrombus. We further assess clinical and pathological factors that may influence survival.

## Materials and methods

**Figure 1:**
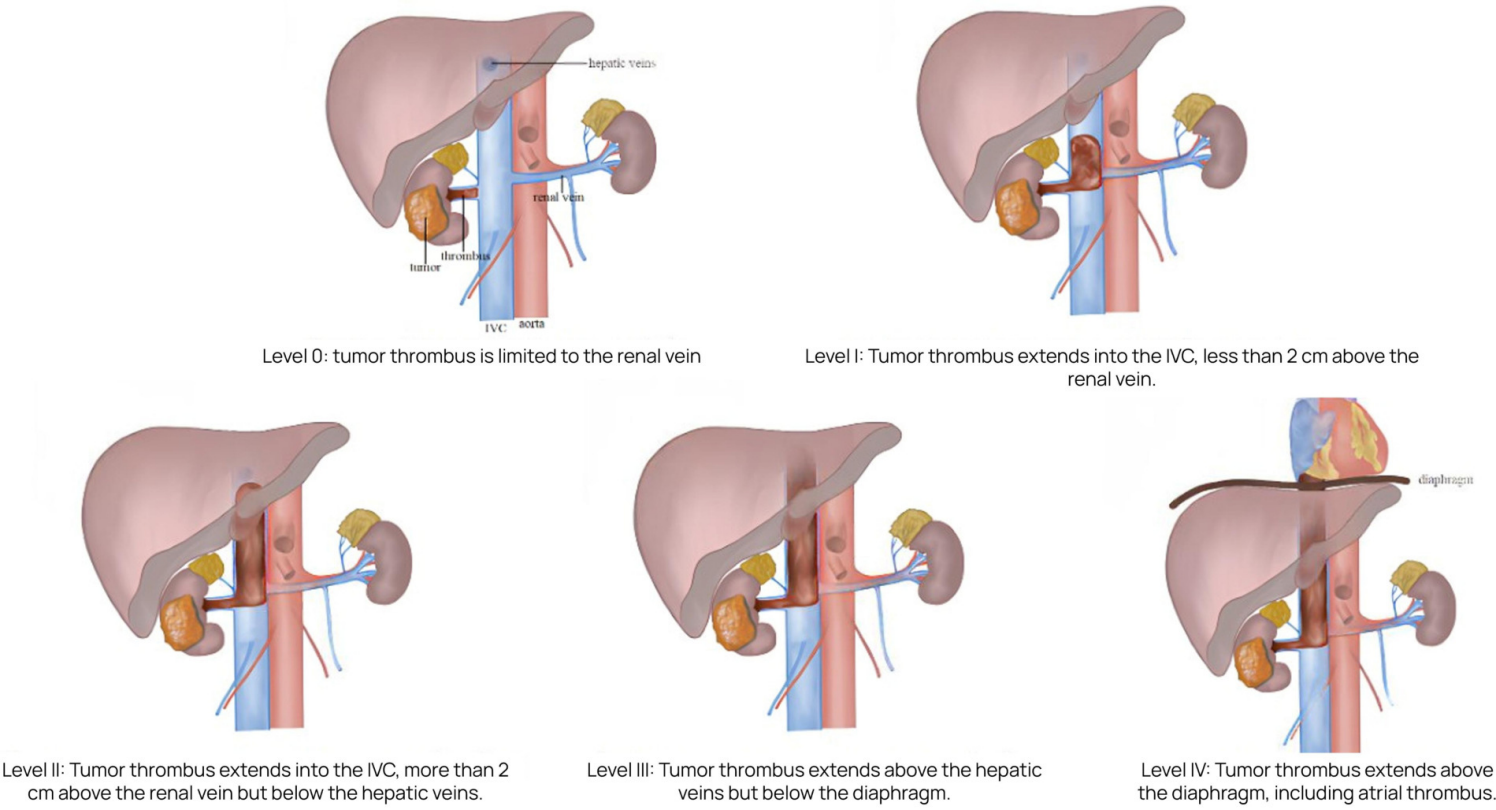
Mayo classification of IVC tumor thrombus in renal cell carcinoma.

We conducted a retrospective analysis of clinical outcomes in 20 patients diagnosed with renal cell carcinoma and tumor thrombus involving the inferior vena cava (IVC) at Mayo levels III–IV (Figure 1). All patients underwent radical nephrectomy with IVC thrombectomy at Botkin Hospital between 2017 and 2024.

**Figure 2:**
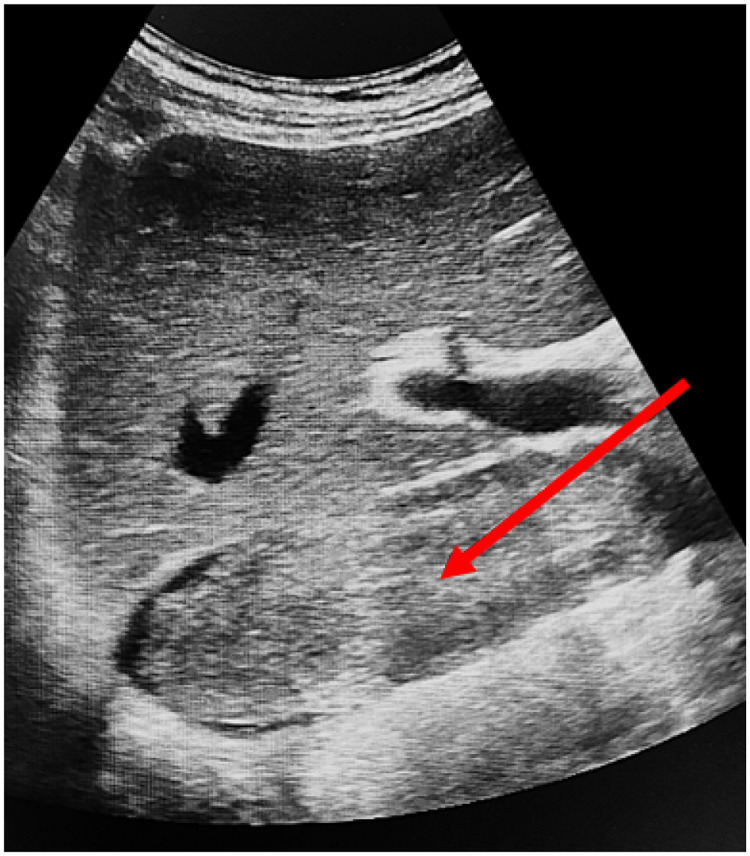
Abdominal ultrasound. Tumor thrombus in the retrohepatic segment of the IVC (red arrow).

**Figure 3:**
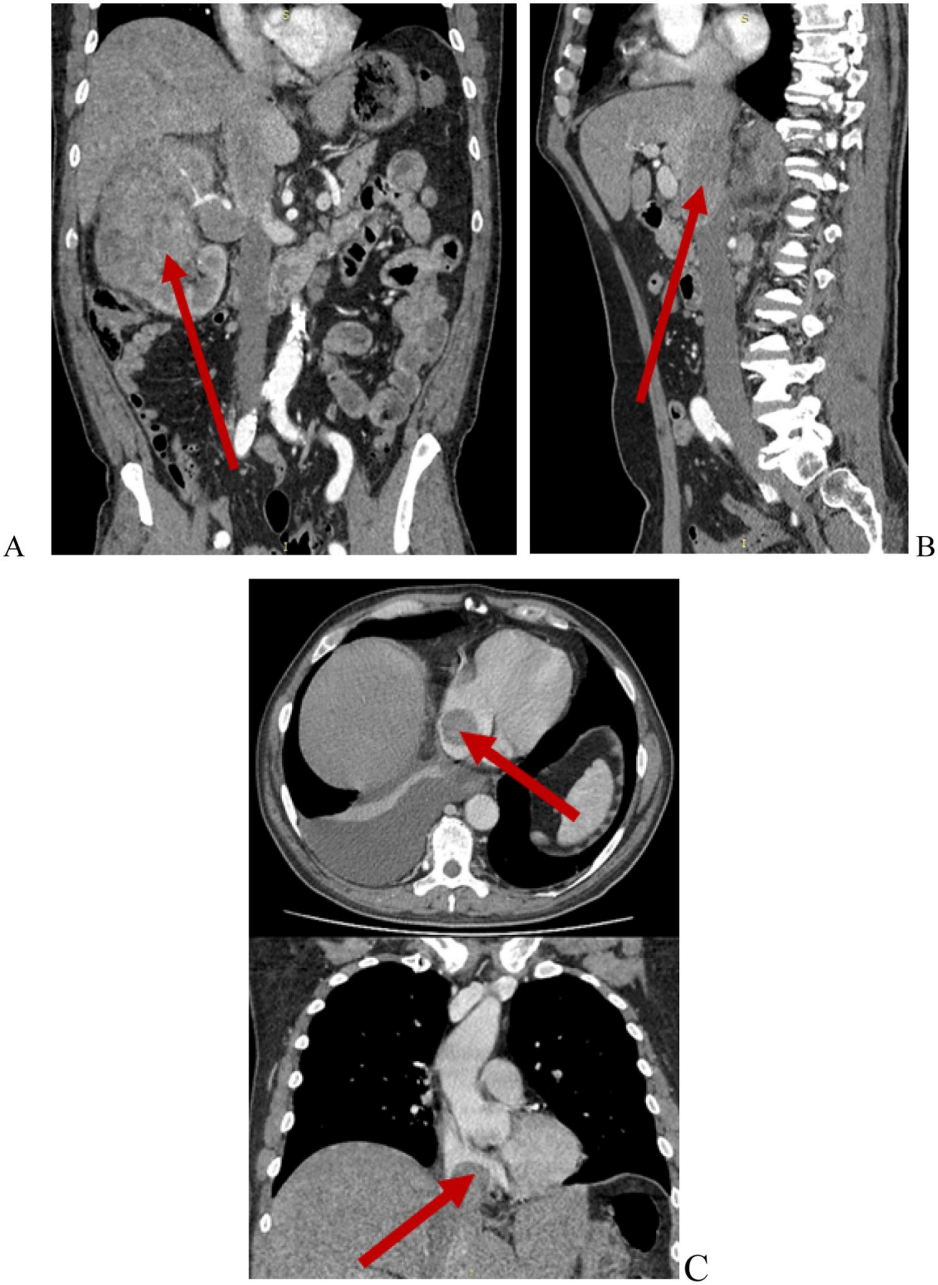
Contrast-enhanced abdominal CT. Renal cell carcinoma with Mayo level IV IVC tumor thrombus. **(A)** Renal tumor (red arrow); **(B)** Tumor thrombus within the IVC and right hepatic vein (red arrow); **(C)** Tumor thrombus extending into the right atrium (red arrows).

Preoperative imaging was performed to determine the optimal surgical approach and the extent of resection. This included magnetic resonance imaging (MRI) using a General Electric Signa Excite 1.5 T scanner, color Doppler ultrasonography with an expert-class GE Logiq E9 system (Figure 2), and contrast-enhanced multidetector computed tomography (MDCT) with a Toshiba Aquilion Prime 160-slice CT scanner (Figure 3). Intravenous contrast was administered using Iohexol (Omnipaque, GE Healthcare). These modalities enabled detailed evaluation of tumor extent and vascular involvement, facilitating precise surgical planning.

**Figure 4:**
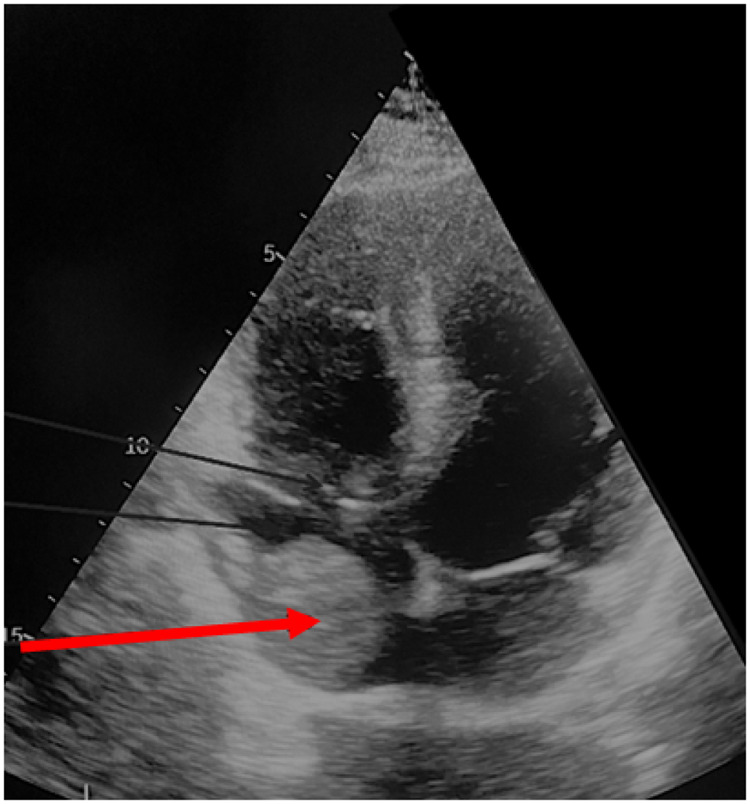
Echocardiogram showing a tumor thrombus in the right atrium (red arrow).

These imaging modalities also facilitated assessment of the oncological stage, the extent and dimensions of the IVC thrombus, as well as the patency of the IVC and collateral circulation. In all patients with level IV tumor thrombus, preoperative evaluation included transthoracic and transesophageal echocardiography to more accurately determine whether the thrombus was adherent to the atrial wall (Figure 4). In such cases, surgery was performed under cardiopulmonary bypass (CPB).

Postoperative complications were classified according to the Clavien–Dindo system. Grade I included any deviation from the normal postoperative course without the need for pharmacological treatment or surgical, endoscopic, or radiological interventions (allowed therapies include antiemetics, antipyretics, analgesics, diuretics, electrolytes, and physiotherapy). Grade II complications required pharmacological treatment beyond that permitted for Grade I, including the need for blood transfusions and total parenteral nutrition. Grade III involved complications requiring surgical, endoscopic, or radiological intervention: IIIa without general anesthesia and IIIb under general anesthesia. Grade IV referred to life-threatening complications requiring intensive care unit (ICU) management: IVa for single-organ dysfunction and IVb for multiorgan dysfunction. Grade V corresponded to patient death (11).

### Surgical technique

All patients underwent nephrectomy, thrombectomy, and extended retroperitoneal lymphadenectomy. Adrenalectomy was additionally performed in 15 cases (75%). A full midline laparotomy was carried out in all cases; in 5 patients, it was supplemented with a median sternotomy. The cranial extent of floating thrombi at Mayo level III–IV and the condition of cardiac chambers following thrombectomy were monitored intraoperatively using transesophageal echocardiography (TEE).

In 5 patients (25%), cardiopulmonary bypass was required. For procedures involving CPB, the surgical protocol began with a complete median sternotomy to ensure immediate access in the event of an emergency initiation of bypass. This was followed by a full midline laparotomy. The ascending colon and duodenum were mobilized using the Cattell–Braasch maneuver to expose the infrarenal segment of the inferior vena cava, the contralateral renal vein, and the affected right kidney. In cases of left-sided tumors, additional mobilization of the descending colon was performed.

In most cases, the renal artery on the affected side was ligated during IVC mobilization—prior to kidney dissection—in order to reduce venous hypertension and minimize blood loss. In cases with significant peritumoral inflammation, the renal artery was accessed separately through the root of the small bowel mesentery, which was particularly important for right-sided tumors.

**Figure 5:**
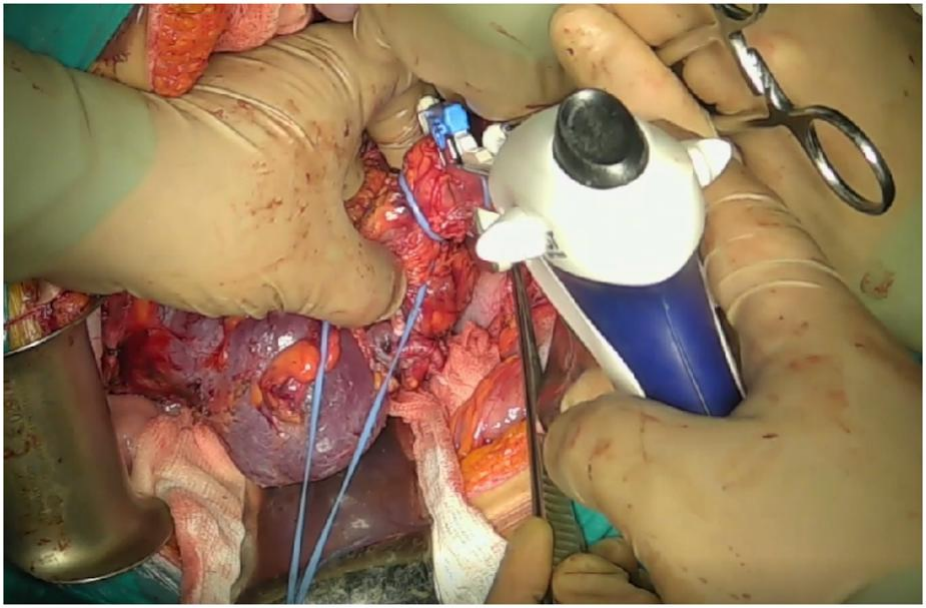
Intraoperative photograph. Stapling of the right renal vein.

Mobilization of the subhepatic IVC was carried out with ligation of the short hepatic veins. This was followed by dissection of the subdiaphragmatic IVC, including ligation of the diaphragmatic veins. To mobilize the retrohepatic segment of the IVC, hepatic ligaments—including the round, falciform, and triangular ligaments—were transected. Nephrectomy was then performed, with renal vein division using a vascular stapler (Figure 5).

To initiate cardiopulmonary bypass, cannulation of the ascending aorta, superior vena cava, and inferior vena cava was performed following systemic heparinization, with activated clotting time (ACT) monitoring.

**Figure 6:**
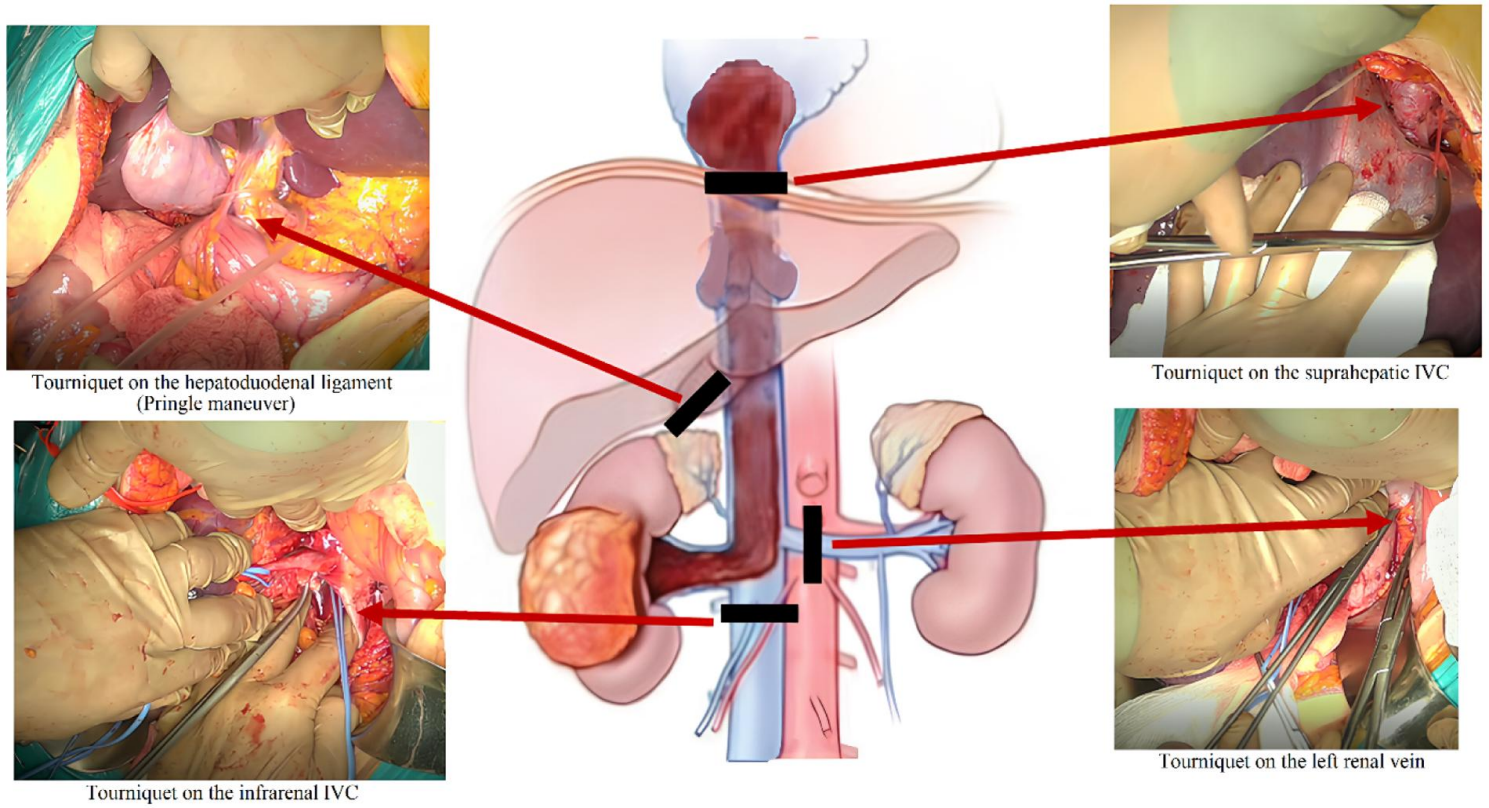
Schematic illustration of the surgical procedure. Clamping of the hepatoduodenal ligament, suprahepatic IVC, contralateral renal vein, and infrarenal IVC prior to thrombectomy.

The cranial portion of the thrombus was extracted under cardiopulmonary bypass via atriotomy. Once complete removal of the thrombus apex from the atrial cavity was confirmed visually, a tourniquet was secured around the subdiaphragmatic segment of the IVC. Following atrial closure, vascular control was achieved from the abdominal field by clamping the hepatoduodenal ligament (Pringle maneuver), the suprahepatic IVC, the contralateral renal vein, and the infrarenal IVC (Figure 6).

**Figure 7:**
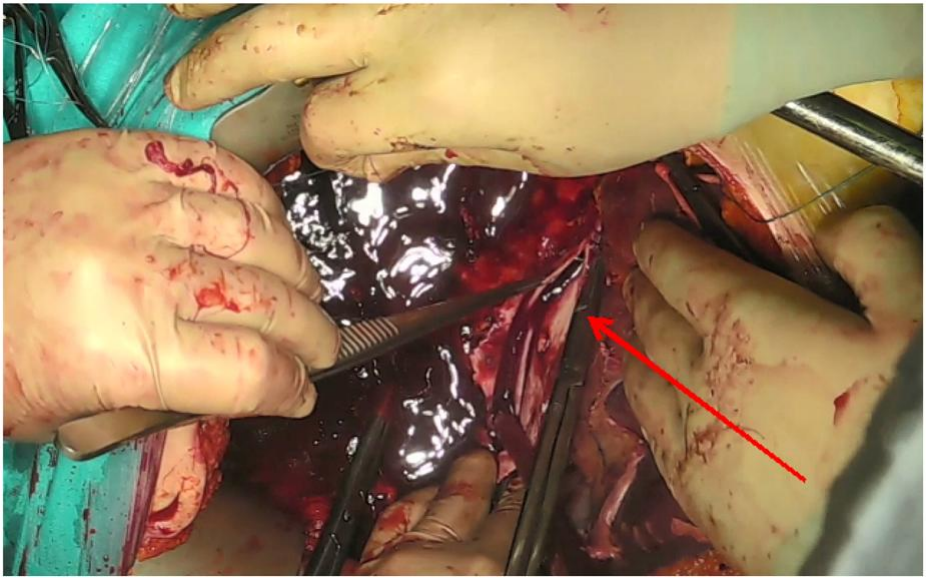
Intraoperative photograph. Primary closure of the IVC (red arrow).

Tumor thrombus removal was performed through a longitudinal venotomy of the IVC. In all cases, the thrombectomy was completed by closing the venotomy site with a single-layer continuous suture (Figure 7). No case required prosthetic reconstruction of the IVC.

In the absence of atrial wall adherence, diaphragmotomy was performed via the abdominal approach to mobilize the intrapericardial segment of the IVC. Under TEE guidance, manual displacement of the thrombus apex from the atrial cavity was achieved, followed by tightening of the tourniquet around the IVC from the abdominal field. The principal stage of the operation was then carried out under vascular control of the IVC and its tributaries. In all cases, a cell salvage system (Cell-Saver) was employed to compensate for intraoperative blood loss.

In several cases, simultaneous procedures were performed, including tumor thrombus removal from the hepatic vein ostia (*n* = 2; 10%), tumor thrombus extraction from the contralateral renal vein (*n* = 3; 15%), and cholecystectomy (*n* = 2; 10%).

### Statistical analysis

Categorical variables were presented as absolute numbers and relative frequencies. Quantitative variables were described by the median and interquartile range (IQR) after assessing the distribution using the Shapiro–Wilk normality test. Kaplan–Meier survival analysis was used to estimate the median overall survival and the 1-year, 3-year, and 5-year survival rates, with corresponding 95% confidence intervals. Spearman's rank correlation analysis was performed to assess the strength and direction of associations between postoperative complications and intraoperative blood loss and operative time. Correlation coefficients (r) were interpreted as follows: 0.00–0.19 as very weak, 0.20–0.39 as weak, 0.40–0.59 as moderate, 0.60–0.79 as strong, and 0.80–1.0 as very strong. A *p*-value of <0.05 was considered statistically significant. The impact of distant metastasis and the administration of immuno-targeted therapy on overall survival was evaluated using univariate Cox proportional hazards regression analysis. Variables with *p* < 0.05 in univariate analysis were considered for inclusion in multivariate Cox models. Hazard ratios (HR) and 95% confidence intervals (CI) were calculated.

## Results

The study cohort included 6 women (30%) and 14 men (70%). The median age was 61 years (IQR: 51–72). Eastern Cooperative Oncology Group (ECOG) performance status was 0–1 in 15 patients (75%), 2 in 3 patients (15%), and 3 in 1 patient (5%).

Right kidney involvement was observed in 15 patients (75%), and left kidney involvement in 5 patients (25%). Tumor thrombus involving the IVC was classified as Mayo level III in 8 patients (40%) and level IV in 12 patients (60%). Clinical signs of IVC syndrome were evident in 2 patients; deep vein thrombosis of the lower extremities was noted in 2 cases, ascites in 1, and pleural effusion in 2. A history of pulmonary embolism involving small branches of the pulmonary artery was recorded in 2 patients with level IV thrombus. Distant metastases from renal cell carcinoma were present in 8 patients (40%), involving one or more organs: lungs (*n* = 4), liver (*n* = 3), and bones (*n* = 1). 11 patients (55%) received immuno-targeted therapy. A detailed overview of the cohort is provided in Table 1.

**Table 1:** Preoperative characteristics of patients.

Parameter	*n* (%)
Sex:	
Male	14 (70%)
Female	6 (30%)
Median age, years	61 (IQR: 51–72)
Side of kidney involvement:	
Left	5 (25%)
Right	15 (75%)
IVC tumor thrombus level:	
Level III	8 (40%)
Level IV	12 (60%)
Distant metastases:	
М_0_	12 (60%)
М_1_	8 (40%)
History of pulmonary embolism	2 (10%)
Immuno-targeted therapy	11 (55%)

There were no cases of intraoperative mortality. The median operative time was 370 min (IQR: 210–660), and the median estimated blood loss was 2,500 mL (IQR: 1,000–4,000). The median volume of autologous blood reinfusion was 750 mL (IQR: 0–2,000). All patients required postoperative care in the intensive care unit (ICU), with a median ICU stay of 3 days (IQR: 1–8). Postoperative complications were observed in 4 patients (20%). Two patients experienced Clavien–Dindo grade IIIA surgical complications: one case of hemopericardium managed with pericardial drainage, and one case of right-sided pneumothorax requiring pleural drainage. In one patient, early adhesive small bowel obstruction developed, necessitating relaparotomy and adhesiolysis (Clavien–Dindo grade IIIB).

Spearman's rank correlation analysis demonstrated a moderate positive correlation between intraoperative blood loss and the occurrence of postoperative complications (r = 0.42, *p* = 0.02), indicating that greater blood loss during surgery was associated with a higher risk of postoperative adverse events. Conversely, operative time showed only a very weak correlation with the incidence of complications (r = 0.11, *p* = 0.54), suggesting that longer operative duration alone did not significantly impact the development of postoperative complications in our cohort. In-hospital mortality was 5% (1/20). One patient developed a subarachnoid hemorrhage on postoperative day 14, requiring neurosurgical intervention. The median length of hospital stay was 11 days (IQR: 8–41). Early postoperative outcomes are summarized in Table 2.

**Table 2:** Surgical outcomes in patients with renal tumors and advanced IVC tumor thrombus.

Parameter	Value
Median operative time, min	370 (IQR: 210–660)
Median blood loss, mL	2,500 (IQR: 1,000–4,000)
Median autologous blood reinfusion, mL	750 (IQR: 0–2,000)
Median ICU stay, days	3 (IQR: 1–8)
Median hospital stay, days	11 (IQR: 8–41)
Postoperative complications (Clavien-Dindo):	
Grade IIIA	2 (10%)
Grade IIIB	1 (5%)
Grade V	1 (5%)

**Figure 8:**
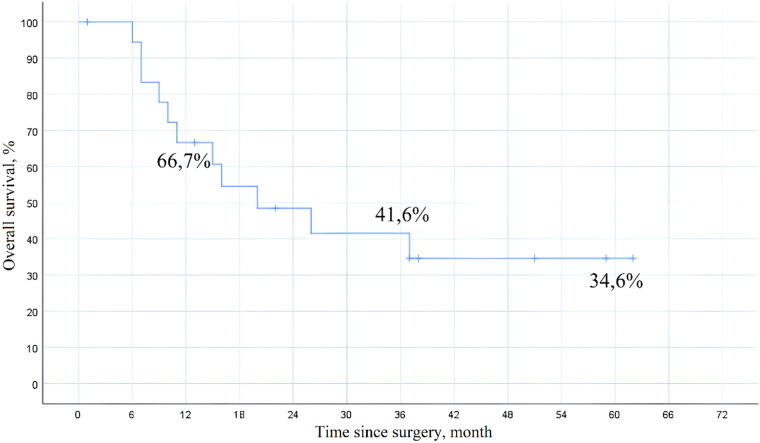
Long-term overall survival following radical nephrectomy and IVC thrombectomy.

Histopathological examination revealed clear cell renal cell carcinoma in 11 patients (55%), while the remaining 9 patients (45%) were diagnosed with clear cell adenocarcinoma. At a median follow-up of 41 months, the 1-year overall survival rate was 66.7%, 3-year survival was 41.6%, and 5-year survival was 34.6% (Figure 8). In the univariate Cox proportional hazards regression analysis, the presence of distant metastasis was significantly associated with decreased overall survival (HR 2.48, 95% CI 1.32–4.65, *p* = 0.005), while patients who received immuno-targeted therapy demonstrated improved overall survival (HR 0.69, 95% CI 0.37–0.85, *p* = 0.035).

## Discussion

Tumor thrombus extension into the inferior vena cava (IVC) in patients with renal cell carcinoma (RCC), particularly at Mayo levels III and IV, presents a significant surgical and oncological challenge. These cases are characterized by an increased risk of massive hemorrhage, thromboembolic events, and complex technical demands associated with mobilization of the IVC and potential intracardiac involvement. Optimal management requires a multidisciplinary approach and careful preoperative planning tailored to individual anatomical and clinical circumstances (12, 13).

In this retrospective series of 20 patients with Mayo level III or IV tumor thrombus, radical nephrectomy with IVC thrombectomy was performed under intraoperative transesophageal echocardiographic guidance. Cardiopulmonary bypass (CPB) was used in 25% of cases. The observed in-hospital mortality rate was 5%, aligning with outcomes reported in other contemporary retrospective cohorts (14–17). The 5-year overall survival rate was 34.6%, which is somewhat lower than previously published data ranging from 40% to 60%. However, this result appears comparable when considering the higher proportion of patients with level IV thrombus (60%) and metastatic disease (40%) in our cohort (15, 18–20).

The findings in our cohort are broadly consistent with previously published series. Chen et al. (2021) reported a 5-year overall survival rate of 39% in 121 patients, among whom 21.5% had level IV thrombus and 35% had metastatic disease (15). Similarly, Gamboa-Hoil et al. (2021) described a 5-year survival of 37% and an in-hospital mortality rate of 4.5% in a cohort of 132 patients, with adverse prognostic features including IVC wall invasion and sarcomatoid differentiation (14). Lambert et al. (2007) reported a 5-year survival of 40.7% in a series of 118 patients, with survival outcomes strongly associated with thrombus extent, disease stage, and lymph node involvement (18). In contrast, Garg et al. (2022) reported a 5-year survival of 63.2% and a median survival of 75 months in a cohort of 56 patients, which was attributed to a more favorable disease profile, including predominantly level I–II thrombi and a lower incidence of metastases (20).

The comparatively lower survival rate in our cohort may be explained by the high proportion of patients with level IV thrombus (60%) and metastatic disease (40%). In univariate analysis, metastatic status was significantly associated with reduced overall survival (HR 2.48; 95% CI 1.32–4.65; *p* = 0.005), consistent with findings from larger international studies (21–23). The relatively short follow-up period (median 41 months) may have also contributed to an underestimation of long-term survival. Since many patients had not yet reached the 5-year mark at the time of analysis, survival estimates were based in part on censored data, which limits their interpretability in the context of a small sample size.

In the surgical management of RCC with level IV tumor thrombus cardiopulmonary bypass (CPB) is often considered necessary to ensure hemodynamic stability and facilitate complete tumor resection. Zacek et al. reported a 5-year survival rate of 51% and in-hospital mortality of 7.9% in patients undergoing CPB-assisted thrombectomy, supporting its use in anatomically complex cases (24).

According to Nesbitt et al., CPB is typically required only when the thrombus extends into the right atrium (25). However, the use of CPB is associated with a range of complications, including acute kidney injury, stroke, coagulopathy, and pulmonary events, highlighting the need for careful patient selection (26, 27).

In selected patients without atrial wall involvement, thrombectomy can be performed without CPB. Several clinical reports have demonstrated that floating thrombi located in the right atrium can be safely managed via an abdominal approach under intraoperative transesophageal echocardiographic guidance, with acceptable oncologic outcomes (28, 29). In such cases, gentle external compression over the right atrium under transesophageal echocardiographic guidance may allow the thrombus to be pushed back into the IVC, enabling safe removal without the need for cardiopulmonary bypass (30).

In our series, CPB was used exclusively in patients with confirmed thrombus fixation to the atrial wall, in accordance with established surgical standards. This strategy provided stable hemodynamics and reduced the risk of tumor fragmentation and embolization during complex thrombectomy procedures. The use of CPB should be based on the anatomical extent of the thrombus and the overall clinical condition. In cases with atrial wall involvement, it remains a justified and safe approach.

Neoadjuvant systemic therapy, including tyrosine kinase inhibitors and immune checkpoint inhibitors, has been investigated as a means of reducing tumor thrombus burden and improving surgical resectability in patients with RCC and IVC involvement. Observational data have suggested that systemic treatment may lead to partial thrombus regression in selected cases (31, 32). However, current guidelines from the European Association of Urology (EAU) consider this approach investigational, and recommend its use be limited to clinical trial settings, as prospective randomized data confirming efficacy and safety are lacking (33).

Segmental resection of the inferior vena cava (IVC) has been employed in cases where tumor thrombus invades the caval wall. Liu et al. reported successful circumferential resections without vascular reconstruction in selected patients, relying on sufficient collateral venous return to maintain hemodynamic stability (34). However, this approach is generally feasible only in cases of right-sided tumors with preserved contralateral renal function (35). The left kidney typically benefits from a more extensive collateral network, whereas the right kidney has limited venous drainage. In such situations, graft reconstruction of the IVC is generally required to preserve adequate venous outflow.

Several histopathological features—such as sarcomatoid differentiation, tumor necrosis, lymph node involvement, and IVC wall invasion—have been independently associated with poor prognosis in RCC patients with venous tumor thrombus (15, 16). In the present study, these variables were not consistently assessed due to the retrospective nature of the data and the lack of centralized pathology review. In several cases, surgery was performed in urgent settings or in patients with advanced tumor burden, which limited the completeness and standardization of pathological evaluation. Considering their clinical relevance, these features should be systematically incorporated into future prospective trials with standardized pathology review.

This study has several limitations. The retrospective design, small sample size, and single-center setting limit the generalizability of the findings. Systemic therapies were administered based on individual clinical decisions rather than standardized protocols, restricting the ability to evaluate their impact on survival. Although univariate analysis identified associations between metastatic disease, systemic treatment, and overall survival, multivariable analysis was not feasible due to the low number of events and missing covariates. Additionally, the absence of centralized pathology review precluded consistent assessment of key histologic features such as necrosis, sarcomatoid differentiation, and IVC wall invasion. Finally, the relatively short median follow-up of 41 months may have affected the accuracy of long-term survival estimates and limited the interpretation of 5-year outcomes.

## Conclusion

Our experience highlights the importance of an individualized surgical approach based on preoperative imaging, assessment of thrombus extent and mobility, intraoperative echocardiographic monitoring, and selective use of cardiopulmonary bypass. Adherence to these principles may help achieve satisfactory oncologic and functional outcomes. Further multicenter prospective studies are warranted to validate prognostic models and to better define the role of neoadjuvant therapy in this high-risk patient population.

## Data Availability

The original contributions presented in the study are included in the article/Supplementary Material, further inquiries can be directed to the corresponding author.

## References

[1] Sung H Ferlay J Laversanne M Soerjomataram I Jemal A Bray F. Global cancer statistics 2020: GLOBOCAN estimates of incidence and mortality worldwide for 36 cancers in 185 countries. CA Cancer J Clin. (2021) 71(3):209–49. 10.3322/caac.21660 33538338

[2] Capitanio U Montorsi F. Renal cancer. Lancet. (2016) 387(10021):894–906. 10.1016/S0140-6736(15)00046-X 26318520

[3] Psutka SP Leibovich BC. Management of inferior vena cava tumor thrombus in locally advanced renal cell carcinoma. Ther Adv Urol. (2015) 7(4):216–29. 10.1177/1756287215576443 26445601 PMC4580091

[4] Blute ML Leibovich BC Lohse CM Cheville JC Zincke H. The Mayo clinic experience with surgical management, complications and outcome for patients with renal cell carcinoma and venous tumour thrombus. BJU Int. (2004) 94(1):33–41. 10.1111/j.1464-410X.2004.04897.x 15217427

[5] Sidiropoulos T Ntella V Margaris I Christodoulou S Theodoraki K Vassiliu P En-bloc resection of renal cell carcinoma with tumor thrombus propagating into the intrapericardial inferior vena cava: efficacy and safety of transabdominal approach. Cureus. (2023) 15(7):e42394. 10.7759/cureus.42394 37621783 PMC10446507

[6] Ciancio G Manoharan M Katkoori D Santos DL Soloway R S M. Long-term survival in patients undergoing radical nephrectomy and inferior vena cava thrombectomy: single-center experience. Eur Urol. (2010) 57(4):667–72. 10.1016/j.eururo.2009.06.009 19560258

[7] Pyrgidis N Schulz GB Stief CG Blajan I Ivanova T Graser A Perioperative outcomes after radical nephrectomy with inferior vena cava thrombectomy. Cancers (Basel). (2025) 17(7):1083. 10.3390/cancers17071083 40227582 PMC11987744

[8] Al Otaibi M Abou Youssif T Alkhaldi A Sircar K Kassouf W Aprikian A Renal cell carcinoma with inferior vena caval extension: impact of tumour extent on surgical outcome. BJU Int. (2009) 104(10):1467–70. 10.1111/j.1464-410X.2009.08575.x 19388993

[9] Ljungberg B Albiges L Abu-Ghanem Y Bedke J Capitanio U Dabestani S European Association of urology guidelines on renal cell carcinoma: the 2022 update. Eur Urol. (2022) 82(4):399–410. 10.1016/j.eururo.2022.03.006 35346519

[10] Dason S Mohebali J Blute ML Salari K. Surgical management of renal cell carcinoma with inferior vena cava tumor thrombus. Urol Clin N Am. (2023) 50(2):261–84. 10.1016/j.ucl.2023.01.007 36948671

[11] Dindo D Demartines N Clavien PA. Classification of surgical complications: a new proposal with evaluation in a cohort of 6336 patients and results of a survey. Ann Surg. (2004) 240(2):205–13. 10.1097/01.SLA.0000133083.54934.AE 15273542 PMC1360123

[12] Yano D Yokoyama Y Tokuda Y Kato M Mashiko Y Kuwabara F Multidisciplinary surgical approach for renal cell carcinoma with inferior vena cava tumor thrombus. Surg Today. (2021) 51(10):1637–44. 10.1007/s00595-021-02415-1 34786640

[13] Tabbara MM González J Ciancio G. Multidisciplinary surgical approach for renal cell carcinoma with inferior vena cava tumor thrombus. Surg Today. (2022) 52(7):1120–1. 10.1007/s00595-022-02528-1 35569082

[14] Gamboa-Hoil SI Hernández-Toríz N Riera-Kinkel C. Outcomes in renal cell carcinoma with inferior vena cava thrombus treated with surgery. Curr Health Sci J. (2021) 47(1):96–100. 10.12865/CHSJ.47.01.15 34211754 PMC8200613

[15] Chen Z Yang F Ge L Qiu M Liu Z Liu C Outcomes of renal cell carcinoma with associated venous tumor thrombus: experience from a large cohort and short time span in a single center. BMC Cancer. (2021) 21(1):766. 10.1186/s12885-021-08508-x 34215223 PMC8254310

[16] Wagner B Patard JJ Méjean A Bensalah K Verhoest G Zigeuner R Prognostic value of renal vein and inferior vena cava involvement in renal cell carcinoma. Eur Urol. (2009) 55(2):452–9. 10.1016/j.eururo.2008.07.053 18692951

[17] Haddad AQ Wood CG Abel EJ Krabbe LM Darwish OM Thompson RH Oncologic outcomes following surgical resection of renal cell carcinoma with inferior vena caval thrombus extending above the hepatic veins: a contemporary multicenter cohort. J Urol. (2014) 192(4):1050–6. 10.1016/j.juro.2014.03.111 24704115

[18] Lambert EH Pierorazio PM Shabsigh A Olsson CA Benson MC McKiernan JM. Prognostic risk stratification and clinical outcomes in patients undergoing surgical treatment for renal cell carcinoma with vascular tumor thrombus. Urology. (2007) 69(6):1054–8. 10.1016/j.urology.2007.02.052 17572185

[19] Berczi A Flasko T Szerafin T Thomas B Bacso Z Berczi C. Surgical management and outcome of renal cell carcinoma with inferior vena cava tumor thrombus. Urol Int. (2017) 99(3):267–71. 10.1159/000464108 28253496

[20] Garg H Nayak B Kumar A Singh P Nayyar R Kaul A Survival analysis and predictors of long-term outcomes following radical nephrectomy with inferior vena cava (IVC) thrombectomy in renal cell carcinoma. Indian J Cancer. (2022) 60(1):127–33. 10.4103/ijc.IJC_5_20 36861688

[21] Choi J Bang S Suh J Choi CI Song W Yuk HD Survival pattern of metastatic renal cell carcinoma patients according to WHO/ISUP grade: a long-term multi-institutional study. Sci Rep. (2024) 14:4740. 10.1038/s41598-024-54052-6 38413653 PMC10899595

[22] Hsieh PY Hung SC Li JR Wang SS Yang CK Chen CS The effect of metastasectomy on overall survival in metastatic renal cell carcinoma: a systematic review and meta-analysis. Urol Oncol. (2021) 39(7):422–30. 10.1016/j.urolonc.2021.02.026 33934963

[23] Pieretti AC Shapiro DD Westerman ME Hwang H Wang X Segarra LA Tumor diameter response in patients with metastatic clear cell renal cell carcinoma is associated with overall survival. Urol Oncol. (2021) 39(12):837.e9–837.e17. 10.1016/j.urolonc.2021.08.013 34551888 PMC9486901

[24] Zacek P Brodak M Gofus J Dominik J Moravek P Louda M Renal cell carcinoma with intracardiac tumor thrombus extension: radical surgery yields 2 years of postoperative survival in a single-center study over a period of 30 years. Front Oncol. (2023) 13:1137804. 10.3389/fonc.2023.1137804 36816971 PMC9931241

[25] Nesbitt JC Soltero ER Dinney CPN Walsh GL Schrump DS Swanson DA Surgical management of renal cell carcinoma with inferior vena cava tumor thrombus. Ann Thorac Surg. (1997) 63(6):1592–600. 10.1016/S0003-4975(97)00329-9 9205155

[26] Ji Q Mei Y Wang X Feng J Cai J Ding W. Risk factors for pulmonary complications following cardiac surgery with cardiopulmonary bypass. Int J Med Sci. (2013) 10(11):1578–83. 10.7150/ijms.6904 24046535 PMC3775118

[27] Huffmyer JL Groves DS. Pulmonary complications of cardiopulmonary bypass. Best Pract Res Clin Anaesthesiol. (2015) 29(2):163–75. 10.1016/j.bpa.2015.04.002 26060028 PMC10068650

[28] Zlatanovic P Koncar I Jakovljevic N Markovic D Mitrovic A Davidovic L. Transesophageal echocardiography-guided thrombectomy of level IV renal cell carcinoma without cardiopulmonary bypass. Braz J Cardiovasc Surg. (2019) 34(2):229–32. 10.21470/1678-9741-2018-0216 30916135 PMC6436781

[29] Komarov RN Rapoport LM Belov YV Germagenova EK Chernyavskii SV Ismailbaev AM Surgical treatment of renal cell carcinoma with tumor thrombosis of the inferior vena cava and the right heart: how we do it. Urologia. (2023) 90(5):470–5. 10.1177/03915603221143566 36803097

[30] Shchukin DV Lesovoy VN Khareba GG Harahatyi AI Maltsev AV Polyakov MM Removal of the tumor thrombus from the right atrium without extracorporeal circulation: emphasis on the displacement of the tumor apex. Adv Urol. (2020) 2020:6063018. 10.1155/2020/6063018 32612649 PMC7320280

[31] Gu L Peng C Liang Q Huang Q Lv D Zhao H Neoadjuvant toripalimab plus axitinib for clear cell renal cell carcinoma with inferior vena cava tumor thrombus: NEOTAX, a phase 2 study. Signal Transduct Target Ther. (2024) 9:264. 10.1038/s41392-024-01990-2 39362847 PMC11450193

[32] Stewart GD Welsh SJ Ursprung S Gallagher FA Jones JO Shields J A phase II study of neoadjuvant axitinib for reducing the extent of venous tumour thrombus in clear cell renal cell cancer with venous invasion (NAXIVA). Br J Cancer. (2022) 127(7):1051–60. 10.1038/s41416-022-01883-7 35739300 PMC9470559

[33] Bex A Abu-Ghanem Y Albiges L Bonn S Campi R Capitanio U European Association of urology guidelines on renal cell carcinoma: the 2025 update. Eur Urol. (2025) 87(4):399–410. 10.1016/j.eururo.2025.03.006 40118739

[34] Liu Z Zhang Q Zhao X Zhu G Tang S Hong P Inferior vena cava interruption in renal cell carcinoma with tumor thrombus: surgical strategy and perioperative results. BMC Surg. (2021) 21:402. 10.1186/s12893-021-01400-2 34802447 PMC8607562

[35] Gonzalez de Gor Herrera V Asencio Pascual JM González J Herranz Amo F LLedó García E Sánchez Ochoa MA Circumferential Inferior vena cavectomy without caval replacement in the management of renal cell carcinoma with tumor thrombus. Curr Urol Rep. (2024) 25(6):117–24. 10.1007/s11934-024-01203-x 38763948 PMC11136755

